# Safe abortion within the Venezuelan complex humanitarian emergency: understanding context as key to identifying the potential for digital self-care tools in expanding access

**DOI:** 10.1080/26410397.2022.2067104

**Published:** 2022-05-20

**Authors:** Génesis Luigi-Bravo, Roopan Kaur Gill

**Affiliations:** a Community Engagement Lead, Vitala Global Foundation, Vancouver, British Columbia, Canada; Graduate Institue of Geneva, Geneva, Switzerland; b Executive Director, Vitala Global Foundation, Vancouver, British Columbia Canada; Clinican Investigator, Assistant Professor, University of Toronto, Department of Obstetrics & Gynecology, Toronto, Canada. *Correspondence:* rgill@vitalaglobal.org

**Keywords:** self-managed abortion, digital health, self-care, humanitarian, sexual and reproductive health, Venezuela

## Introduction

Fulfilling sexual and reproductive health and rights (SRHR) in Venezuela is a complex challenge. A Venezuelan woman needs more than 15 times the average monthly salary to buy a month’s worth of contraceptive pills, and she would also have to go to several pharmacies or online vendors to find them. Due to chronic supply stock-outs, many people have to resort to clandestine markets or high-end pharmacies to find sexual and reproductive health (SRH) supplies.^1^ These shortages include other SRH essentials such as condoms for preventing sexually transmitted infections, pads for menstrual health management, and the full contraception mix to prevent unwanted pregnancies. Many of these SRH supplies are considered luxury items in Venezuela. Their inaccessibility exemplifies how complex humanitarian emergencies deepen socioeconomic disparities in the free exercise of bodily autonomy and sexual and reproductive rights.

The World Health Organization [WHO] defines “complex humanitarian emergencies” as significant disruptions in livelihoods, the product of a combination of political instability, violence, social inequities, and underlying poverty that lead to large-scale displacements.^2^ Several human rights organisations have used this term to define the Venezuelan situation, given the complexity of the political crisis that turned the country’s economy into one of the most volatile in the world.^3,4^ Causes of the crisis include the mismanagement of foreign exchange reserves and national budgets, the decline of oil revenues in 2013, and the international economic sanctions in 2017.^5^ As a result of this situation, in 2020, many people earned a monthly minimum wage equivalent to US$ 1.50 that was continuously losing value, with the cumulative yearly inflation rate reaching 3,713%.^6,7^ Consequently, more than half of the Venezuelan population live in conditions of extreme poverty, or heavily dependent on remittances in $US.^6^ This situation has led many to migrate in order to access essential public services, reliable water and sanitation services, nutrition, and health care, including maternal and child health, as well as SRH.^8^

Women’s, adolescents’ and young girls’ health is disproportionately affected by the crisis. In terms of SRH indicators, in 2019, UNFPA reported 95 births per 1000 women aged 15–19, a figure considerably higher than the regional average for Latin America (62 per 1000 women aged 15–19).^9^ This adolescent fertility rate coexists with one of the highest maternal mortality rates in the region (125 deaths per 100,000 live births).^10^ Data gathered by civil society suggests that 13% of all maternal mortality in Venezuela is due to unsafe abortion.^1^ The SRHR context has also driven a significant number of women to migrate or temporarily move to nearby countries to give birth, obtain safe abortions, buy contraception or flee from gender-based violence.^11^ Nevertheless, this is a far from sustainable solution since it relies on forced displacement. It does not improve existing healthcare services and livelihoods in Venezuela, which would enable these women to freely exercise their sexuality in a safe environment.

In this context, Venezuelans have been turning increasingly to digital tools to find information, supplies, and healthcare services, including SRH.^12^ Digital health is an umbrella term that refers to the use of information and communication technologies for health to advance access to universal health coverage.^13^ According to Váyalo Foundation, 51% of adolescents and young people in Venezuela use the internet and social media as primary sources to access SRH information.^14^ In addition, a diverse network of abortion hotlines in Venezuela provides information on safe medical abortion and virtual accompaniment through digital means (phone and instant messaging platforms), and the demand is considerable. In 2019, the hotline Faldas-R received a total of 1142 calls with information requests.^15^ Digital self-care tools could have a prominent role in facilitating information provision and strengthening accompaniment models, considering that one of people’s first contacts with SRH content, including abortion, is through the web or mobile devices.^14,15^

This commentary aims to highlight the importance of understanding the context when designing digital tools for SRH self-care in humanitarian settings. We draw insights from the contextual analysis that was essential to the design process of Aya Contigo, a digital self-care tool that provides information and virtual accompaniment during the medical abortion process up to the first trimester. The overall research and design process included the following phases: (a) contextual analysis and stakeholder engagement and mixed-method exploratory research, (b) user-centred design principles to design and develop the mobile-based digital tool, and (c) mixed-methods feasibility and acceptability pilot study. We focus on findings from the exploratory research. In this phase, we conducted a contextual analysis through a desk review of Venezuela’s complex humanitarian emergency; a mapping of national SRH and abortion legislation, policies, programmes, and main stakeholders; and in-depth interviews with local activists, SRH service providers, and prospective users. We also facilitated workshops where stakeholders shared their inputs on the pertinence of digital self-care tools in the Venezuelan context.

The main takeaway from the contextual analysis and stakeholder interviews was that community members (grassroots organisations, collectives, local providers, and prospective users) must be at the centre of each digital SRH self-care development phase. Digital health implementers must acknowledge that these tools function within an ecosystem of already existing networks of care, providers, and community activists that deliver SRH information and services. Likewise, the overall research and design process of digital health tools should be flexible to adapt to the rapidly changing environment of humanitarian settings, which often have poor communications infrastructure and electrical supply, deficient public transportation to mobilise, and a political environment that hinders open advocacy for reproductive justice, including abortion rights.^1,16^

## Sexual and reproductive health and abortion access in Venezuela’s complex humanitarian emergency

Globally, in 2020, about 34 million women of reproductive age (15–49 years old) were in need of humanitarian assistance to cover not only shelter, clean water, and food but also primary health care that includes SRH services.^17^ Humanitarian settings are contested spaces when it comes to guaranteeing access to these services, especially abortion. Even though abortion is part of essential healthcare protocols such as the minimum initial service package (MISP), which aims to serve as an initial point for SRH programming to save lives, prevent illness, disability, and death in humanitarian settings,^18^ it remains neglected, due to different factors like a perceived lack of need, inadequate protocol implementation, and restrictive legislation.^19^ Although indicators for mortality and morbidity due to unsafe abortion in humanitarian settings are hard to determine, several studies show that of the estimated 73 million abortions happening each year, half occur in unsafe conditions and cause between 8% and 11% of almost entirely preventable maternal deaths.^20^ Furthermore, about 97% of unsafe abortions take place in low- and middle-income countries (LMICs), where humanitarian emergencies are more likely to occur.^21^ Additionally, LMICs are more likely to have restrictive abortion laws, leaving those who need to terminate a pregnancy with a high unmet need for comprehensive abortion and post-abortion services.^21^ Systematic reviews also evidence non-systematic implementation of the MISP related to abortion, contraception, and adolescents’ health in humanitarian settings.^22–24^

Venezuela is no exception to this generalised neglect of abortion services in humanitarian settings. With a healthcare system that has been rapidly deteriorating since 2013, many public hospitals and points of care cannot provide a safe space for abortion seekers.^25^ This service deficiency is due to legislation that only allows abortion to “save the mother’s life”^26^ [p68], a lack of skilled healthcare professionals, SRH supply chain disruptions, and chronic hospital infrastructure failure, evidenced by interruptions in running water and electrical supply.^27^ Meanwhile, many abortion seekers, especially adolescents, cannot afford the high prices of the private sector for clandestine abortions and other SRH services. These precarious conditions contributed to an increase of 66% in the maternal mortality indicators from 2015 to 2016^1^ on current estimates, with no reliable official data. The Venezuelan Ministry of Health has not published official epidemiological reports on maternal health since 2017.^28^ Data collected by civil society organisations estimate that 16% of maternal deaths in the country result from unsafe abortions. However, this figure can increase by up to 60% in rural and indigenous communities.^1,27^ While abortions can be safe when the proper protocols and quality of care are in place to allow abortion seekers to exercise their reproductive autonomy, this panorama illustrates how a number of abortions remain unsafe in humanitarian settings like Venezuela due to structural and institutional barriers.

Under circumstances of restrictive abortion legislation, poor healthcare infrastructure for obstetric care, and limited coverage and quality of SRH services to prevent unwanted pregnancies, the need for safe termination of pregnancy options increases.^29^ Furthermore, providing safe abortion care and information in humanitarian settings is not only a matter of public health outcomes, but also a matter of protecting human rights. According to testimonies from our contextual analysis interviews with key stakeholders, many Venezuelan women face sexual and reproductive coercion. For instance, many women are forced to continue unwanted pregnancies, and young women opt for tubal ligations because it is the only contraceptive option available in many healthcare facilities.^30^ These forms of reproductive coercion are considered torture and a major violation of Venezuelan women’s human rights.^31^

## Self-managed medical abortion: an alternative to exercise reproductive autonomy in humanitarian settings

According to the WHO, self-managed medical abortion is a safe procedure that offers a non-invasive and acceptable option to terminate a pregnancy during the first trimester by using pills (misoprostol alone or in combination with mifepristone) outside the formal healthcare system.^32,33^ Medical abortion as a self-care intervention becomes especially relevant when more than half of abortions happen outside of a health facility.^34^ This figure might be significantly higher in Venezuela, given the highly restrictive legal framework and the chronic shortage of SRH supplies and services to prevent unwanted pregnancies.^26^ In the current humanitarian emergency, self-managed medical abortion can be an alternative to less safe options like clandestine in-clinic procedures with unskilled providers.^35^

However, acquiring abortion medication in Venezuela is a complex endeavour. Even though misoprostol is registered in the country, its purchase remains highly inaccessible and widely stigmatised.^36^ According to the desk review and data from interviews, most people buy the pills online through informal vendors who offer no quality controls, price regulations, or accurate usage information. The illicit nature of this exchange exposes abortion seekers to counterfeit medication or getting the wrong amount of pills.^37^ In addition to this, until July 2020, the misoprostol dosage cost for one first-trimester abortion ranged from US$ 80–200, without allowing for sanitary pads for bleeding management, pain medication, or extra dosages if necessary.^37^ This amount is undoubtedly restrictive, given that a substantial out-of-pocket expenditure would be required to obtain the pills. Nevertheless, medical abortion remains more affordable than in-clinic procedures that cost from US$ 100–500, according to our interviewees.

Due to profound health inequities, self-managed medical abortion in Venezuela acts as a tool for harm reduction but also as an act of resistance to reproductive injustice.^38^ Despite the restrictive abortion legislation, a diverse network of feminists, reproductive justice activists, abortion accompaniers, and health providers offer information on safe medical abortion through digital means like hotlines, instant messaging platforms, and social media.^39^ These community-based organisations play a crucial role in providing evidence-based information and accompaniment.^40,41^ This community-based strategy is a way in which abortion seekers can have safer procedures at home or in safe places of their choice. This model of care is not new; it has already been documented that hotlines can provide high-quality abortion care in contexts like Venezuela, where legal restrictions intersect with humanitarian crises.^40,41^

Digital self-care tools specially designed to facilitate information on medical abortion could expand and optimise access to accurate guidance on how to use the abortion medication safely.^40,42^ These tools can strengthen the essential work of hotlines, accompaniers, and healthcare providers in Venezuela who are implementing harm and risk reduction, post-abortion care, and feminist accompaniment protocols in their communities.^15,39^ Digital self-care innovation could also potentially reach more abortion seekers in hard-to-reach areas where they may not immediately go to a healthcare facility.

## Aya Contigo: a digital self-care tool for medical abortion

Digital health is a broad field that uses information and communication technologies for health.^13^ Evidence has suggested that digital tools can increase SRH information, improve the correct use of interventions, and empower individuals to manage their health and realise their sexual and reproductive rights.^35,43,44^ In the specific case of medical abortion, the use of digital health tools such as safe abortion hotlines and telemedicine models of care has a long history.^38,40–43^ Over the last decade, there has been an increase in the outreach of these tools through using mobile-based texting, calls and, social media to provide harm reduction counselling for medical abortion, emotional support, referrals, and even other SRH services.^38,40–43,45,46^ Furthermore, during the global COVID-19 pandemic, it has become even more apparent that digital health can provide a reasonable and feasible alternative that does not compromise the quality of care and can help connect people to trusted healthcare providers.^33,47^ In Venezuela, the use of digital tools for self-managed medical abortion is palpable in the rich network of hotlines, collectives, and communities of care that provide safe abortion information and care that otherwise might not be available.^15,39,48^

During this first phase of exploratory research, Aya Contigo’s research team conducted a contextual assessment that included: a desk review of current civil society and government reports and literature about SRH in Venezuela; a mapping of key actors, organisations, and local collectives aimed at the promotion and provision of SRH services; and a series of in-depth interviews and group meetings with these stakeholders. This process was crucial to understanding how Venezuelans interact with digital tools for health and to identifying the challenges they face when looking for contraception and abortion information and services. For example, while unstable internet, high cost of phone data, limited access to modern smartphones, and web censorship emerged as barriers that prevent many abortion seekers from accessing information, hotlines, and potential providers, digital resources are extremely useful when an online connection is available, and the internet is one of the leading referral channels used by safe abortion hotlines to connect with their users. Digital innovations are thus an appropriate self-care intervention with the potential to demonstrate a virtual accompaniment model as a safe, acceptable, and feasible tool to support users’ quality of care through a self-managed medical abortion.

This contextual assessment enabled us to identify and understand how these barriers impact access to SRH care as well as the dynamics of the existing networks of hotlines and abortion accompanier collectives in Venezuela. During interviews, participants mentioned that a tool like Aya Contigo could help expand the outreach of safe abortion information and strengthen their accompaniment models, since their work is volunteer-based and demand sometimes exceeds their capacity to take care of all the requests promptly. This feedback encouraged the design team to include within Aya Contigo information that helps users identify gestational age, their eligibility for a medical abortion, how many pills they need, and assess the abortion outcome. This information is compatible with the resources and accompaniment given by hotlines. Similarly, it led the team to include a list of resources to connect with trusted local providers for other SRH needs like contraception or counselling.

Having an exploratory research process that included a diversity of stakeholders ranging from SRH providers to safe abortion activists has been an essential element of this research and design process. The process enabled us to ensure prospective users are represented and that the information shared through Aya Contigo is medically accurate, responds to the needs of abortion seekers, is accessible, easy to understand, protects users’ privacy, and is free from stigma. Digital self-care interventions are not exempt from complying with quality of care guidelines, and all the more so if they are being implemented in humanitarian settings where abortion is legally restricted, and issues like privacy are a priority.^35,43,49^

As with any other self-care intervention, digital tools for self-managed abortion do not work in a vacuum. They function and develop within an ecosystem of different stakeholders, institutions, policies, and legislation.^44^ Engaging community members and key stakeholders in the design process is thus not only a technical requirement but a political commitment to the networks of care already working on expanding access to abortion in humanitarian settings like Venezuela. In this sense, a community-driven approach to digital health brings a breadth of opportunities and challenges that may make the research and design process for the tools more complex, but represents the only way to guarantee that high quality of care is present outside the clinical setting.

## Concluding remarks

Abortion, apart from being a healthcare service, is a community-bounded process^38^ and, as such, is heavily influenced by the sociopolitical environment and local legislation. Working towards increasing access to high-quality abortion care through digital self-care tools requires a careful understanding of the context, especially in humanitarian settings where SRH needs are often neglected, and the adoption of digital self-care innovations faces several implementation challenges. For instance, in Venezuela, reproductive injustice is accentuated by a complex humanitarian emergency that prevents people from exercising their rights to health, reproductive autonomy, and dignified livelihoods. Considering the complexity of the crisis, identifying, partnering, and building trust with local stakeholders throughout the research and design processes is critical. This community engagement is an essential part of having a comprehensive understanding of the SRH landscape and how people navigate it. Furthermore, having an in-depth knowledge of both the context and community needs strengthens the potential of digital self-care interventions to support the delivery of pertinent and high-quality abortion services.

From this first exploratory research phase, our contextual analysis shows that more formative research is needed to identify improvement points and challenges for digital SRH self-care implementation in humanitarian settings.^35^ Including diverse community members in the design and research process of digital SRH self-care interventions is necessary to identify, map, and understand the unique SRH needs of populations like youth and adolescents, persons with disabilities, people with diverse sexual orientations and gender identities and expressions, as well as those who are from indigenous communities, or live in rural areas. Similarly, it is necessary to deepen our understanding of how individuals from these groups access and interact with information about abortion. This further research will help identify challenges and opportunities in designing digital self-care tools that are true to inclusive and intersectional values and practices.

We acknowledge that digital health innovations for abortion access are far from being a quick fix to all the burdens and human rights violations abortion seekers endure in their journey to exercise their reproductive autonomy, especially in humanitarian settings. Digital tools for abortion self-care like Aya Contigo expand the toolbox. Communities and networks of care can take ownership of these digital innovations to strengthen their accompaniment models by emphasising the empowerment of abortion seekers through self-care practices.^38^ Finally, we envision digital self-care not as a replacement to comprehensive clinical care, but as an opportunity to explore new and alternative models of abortion care.^40,41,47^
